# Trends and Levels in Men’s and Women’s Fertility Goals in the United States

**DOI:** 10.1007/s11113-025-09989-5

**Published:** 2026-01-22

**Authors:** Luca Badolato, Sarah R. Hayford

**Affiliations:** Ohio State University, Columbus, USA

**Keywords:** Childbearing, Childlessness, Gender differences, Postponed fertility, Unintended fertility

## Abstract

Understanding trends in fertility goals (attitudes, desires, intentions, etc.), as well as variation by age and parity, is important for understanding current U.S. fertility and assessing likely future outcomes. Both men’s and women’s childbearing goals shape fertility behavior. However, most research on fertility goals focuses on women, and little is known about how men’s fertility goals may have changed over time or vary by age and parity. In this paper, we draw from the U.S. National Survey of Family Growth 2011–2019 to estimate trends in age- and parity-specific indicators for both men and women of (i) the proportion of positive prospective fertility intentions, (ii) the timing of prospective fertility intentions, and (iii) the retrospective reporting of fertility desires. Results show important differences and similarities in men’s and women’s fertility goals, as well as a mixed picture regarding gender convergence or divergence in fertility goals, depending on the exact outcome analyzed. Men are more likely to intend a(nother) child and have greater intentions to delay childbearing, both at the aggregate and across age and parity. Prospective intentions declined for both men and women, but at a higher rate for women, and the declines were proportionally larger early in the life course. For both men and women, we find increases in intended childlessness and intentions to delay childbearing. These two processes together point to potential future declines in cohort fertility, both through unrealized fertility and voluntary childlessness. We conclude by discussing the benefits and challenges of including men in fertility research.

**Supplementary Information:**

The online version contains supplementary material available at 10.1007/s11113-025-09989-5.

## Introduction

In contemporary low-fertility regimes, childbearing is understood to be a matter of individual decision-making, and fertility goals (an umbrella term including fertility attitudes, desires, intentions, and other related constructs) are a central element in understanding fertility behaviors. In particular, the sustained declines in fertility rates that have been observed across high-income countries since 2008 (Comolli, 2017; Sobotka et al., 2011, 2023) have spurred renewed attention to childbearing desires and intentions. To date, the role of changing desires and intentions for children in explaining these downturns is unclear, with some recent research suggesting that fertility goals have been largely stable and declines have been driven by postponement of childbearing and unrealized fertility goals (Guzzo & Hayford, 2023; Hartnett & Gemmill, 2020; Kost et al., 2023).

Much, though not all, recent research on fertility goals and unrealized fertility focuses on women (but see Bozick, 2023; Guzzo & Hayford, 2023; Hartnett & Gemmill, 2020; Nitsche & Hayford, 2020). In particular, little is known about gender differences in the quantum and timing of prospective fertility intentions, in the assessment of retrospective fertility desires, and in variation by age and parity in fertility goals. Because current declines in period fertility are largely driven by fertility postponement and timing changes (Wu & Mark, 2023), age- and parity-specific measures of fertility goals are particularly important for understanding how these period changes may (or may not) be translated into declines in future completed cohort fertility.

In this paper, we address this gap by estimating levels and trends in age- and parity-specific indicators of fertility goals in the United States for both men and women. We draw on data from 2011 to 2019 of the National Survey of Family Growth (NSFG), the most comprehensive nationally representative repeated cross-sectional survey of U.S. fertility goals and behaviors, and estimate aggregate and age- and parity-specific indicators of (i) the proportion of positive prospective fertility intentions, (ii) the timing of prospective fertility intentions, and (iii) the retrospective reporting of fertility desires. The inclusion of men’s goals in these detailed descriptions of trends is a key contribution of this paper. We conclude by discussing how these analyses contribute to our understanding of differences between men’s and women’s fertility goals, fertility decline, and future fertility trends, and by highlighting the benefits and challenges of including men in fertility research.

## Fertility Goals and Behaviors in Contexts of Declining Birth Rates

In general, while fertility rates have continued to decline across low-fertility countries over the past several decades, levels of ideal and intended fertility have been relatively stable. As a result, there is a gap between fertility goals and realized fertility both at the individual and the aggregate level (Harknett & Hartnett, 2014; Morgan & Rackin, 2010; Nitsche & Hayford, 2020; Sobotka & Beaujouan, 2014). Recent research in the United States shows only a marginal decline in total intended parity after the Great Recession (Hartnett & Gemmill, 2020), an increased likelihood of not achieving or revising early-life fertility goals (Guzzo & Hayford, 2023), and increasing rates of pregnancies reported occurring later than wanted among women in their late thirties (Kost et al., 2023). Taken together, these findings suggest that fertility delays and declines may be partly driven by constraints, rather than goals, and point to the importance of considering the timing as well as the quantum of fertility goals and outcomes.

Both men’s and women’s fertility goals influence childbearing behavior (Schoen et al., 1999), but most research on childbearing intentions and desires is limited to women. Existing research on fertility goals that has included both men and women focuses on different aspects of prospective goals and realization. For example, Guzzo and Hayford (2023) analyzed achieved parity, intended parity, underachieved parity, and fertility desires and intentions at 40–44 in the United States; Harnett and Gemmill (2020) studied intended parity, aggregating men and women in most analyses to describe changes in the U.S. population as a whole; and Nitsche and Hayford (2020) examined the correspondence between early fertility desires and achieved fertility in a single U.S. cohort that completed childbearing in the early twenty-first century. The limited research that has specifically focused on men’s fertility goals suggests that childbearing is becoming less important among U.S. young men, which could help explain declining fertility rates (Bozick, 2023).

### Gender Differences in Fertility Goals and Behaviors

The lack of data and research on men’s fertility goals reflects the historical focus on women’s fertility in demographic analyses and data collection. As portrayed by Greene and Biddlecom (2000, p. 81), despite men’s theoretical importance in fertility research, “the assumption of women’s primacy in fertility and contraceptive use has led to a downplaying of men’s roles in studies of fertility and family planning.” When men are included in fertility studies, they are often framed as “problematic” and posing obstacles to women’s reproductive autonomy (Greene & Biddlecom, 2000). More than two decades after Greene and Biddlecom’s article was published, progress has been made in providing basic descriptions of fertility quantum and timing for men, but substantial gaps in knowledge remain.

A small body of research has focused on estimating men’s fertility behaviors (Coleman, 2000; Dudel & Klüsener, 2016; Rampazzo et al., 2018; Schoumaker, 2019; Zhang, 2011). For the most part, this literature is not expansive enough to make systematic comparisons across contexts; we include research from the United States as well as from other settings in this review. The mean age at fatherhood is higher than the mean age at motherhood across all observed countries, and men show a higher variance in mean age at childbearing than women (Billari et al., 2007; Schoumaker, 2019). Men’s and women’s fertility rates, given differences in age-specific fertility and mortality rates and skewed population age structures, can differ substantially, especially at the onset of the fertility transition (Schoumaker, 2019).

In addition to higher fertility rates at older ages, men perceive less strict biological and normative limits for childbearing than women (Billari et al., 2007, 2021; Johfre & Saperstein, 2023; Wagner et al., 2019). Revisions in fertility intentions across the life course differ between men and women, with men being more influenced by their partner’s age and more likely to revise their fertility intentions upward after repartnering, while women are more influenced by their own employment status and income (Guzzo, 2014; Iacovou & Tavares, 2011). Across low-fertility countries, women report more positive attitudes than men toward voluntary childlessness, especially when highly educated, employed, and when reporting less traditional attitudes about marriage (Koropeckyj-Cox & Pendell, 2007; Merz & Liefbroer, 2012). These differences might reflect higher opportunity costs for motherhood than fatherhood driven by uneven structural opportunities and constraints (Glauber, 2018; McQuillan et al., 2008). At the same time, women who decide not to have children are more likely to be stigmatized and devalued compared to men and are more often the target of political attacks and everyday interactions portraying them as selfish (Blackstone, 2019; Hintz & Tucker, 2023; Park, 2002).

On a parallel line of inquiry, research highlights different returns of childbearing for men and women, with mothers reporting lower levels of life satisfaction and happiness than fathers, particularly in countries with higher levels of gender inequality and lower formal welfare support (Aassve et al., 2012, 2015; Margolis & Myrskylä, 2011). In the workplace, motherhood can result in wage penalty, workplace discrimination, job loss, and involuntary unemployment, while men experience a “fatherhood premium” in wages (Byron & Roscigno, 2014; Correll et al., 2007; Glauber, 2018; Yu & Hara, 2021). At home, women continue to spend more time on housework and childcare than men, although these differences are shrinking, and gender differences in housework widen after the birth of a child (Milkie et al., 2025; Yavorsky et al., 2015). Emerging research on fatherhood provides a critical background on the different lived experiences of fathers and mothers (Palkovitz & Hull, 2018; Randles, 2020; Schoppe‐Sullivan & Fagan, 2020). Taken together, this research suggests that men and women may have different fertility goals developed in response to different anticipated experiences of parenthood. To the extent that gender differences in experiences of parenthood are shrinking, as suggested for example by convergence in the amount of time fathers and mothers spend with children, fertility goals may also be growing more similar for men and women.

### Present Study

Despite this relatively rich sociological and demographic background suggesting that men and women might have different childbearing attitudes and goals across the life course, analyses of men’s fertility goals are rare. The overarching aim of this analysis is to provide a comprehensive descriptive picture of men’s and women’s fertility goals in the United States in order to assess similarities and differences, both in trends and in the patterning of quantum and timing goals. This rich description is intended to serve as a foundation for future research focused on particular comparisons and explanations, as well as providing additional evidence on the question of how changing fertility goals may be contributing to ongoing fertility declines. Toward this overarching aim, we pose several specific research questions inspired by existing research.

*RQ1: Has the quantum of intended fertility declined over time for men and women, and if so, has it declined at the same rate for men and women? Do trends vary by age or parity?*

Existing evidence shows that there has been limited decline in aggregate-level measures of women’s fertility goals. Based on this evidence, scholars generally conclude that changing goals have played a limited role in explaining declining birth rates. We examine men’s as well as women’s fertility intentions, compare trends for men and women, and study trends by age and parity in order to better understand how fertility goals may have changed.

*RQ2: Are men more or less likely to intend to have no children than women, and is the prevalence of intended childlessness converging, diverging, or remaining stable between men and women?*

Women hold more positive attitudes toward voluntary childlessness than men, but also are more stigmatized for choices not to have children. We describe patterns of intended childlessness among men and women and trends over time to understand how these conflicting experiences translate into individual goals and whether gender differences are converging, diverging, or remaining stable.

*RQ3: Do men have later desired and intended fertility than women, and do trends in the timing of desired and intended fertility differ for men and women?*

On average, men have children at later ages than women. We expect intentions to reflect this pattern, with men intending longer delays in childbearing and, potentially, being more likely to retrospectively report births as taking place earlier than desired than women. Considering fertility postponement, and given that research in other low-fertility settings has found that the ideal age at parenthood increased at similar rates for men and women (Billari et al., 2021), we expect that intentions to delay childbearing increased at similar rates for men and women.

By addressing these three questions, we provide insight into the possible contribution of men’s fertility goals, both quantum and timing, to declining fertility rates. In particular, we provide important baseline indicators to better understand levels and trends in fertility postponement and voluntary childlessness, two potentially important mechanisms to predict future fertility trends. We also provide insights into the life course patterning of men’s and women’s fertility goals across age and parity.

## Data and Methods

This study is based on data from the National Surveys of Family Growth (NSFG), the most comprehensive nationally representative fertility survey in the United States. The NSFG is a repeated cross-sectional survey started in 1973 to produce national estimates of a broad range of fertility-related indicators, including desires and intentions for children as well as childbearing outcomes. Originally designed to include only women aged 15 to 44, in 2002 the NSFG added an independent sample of men aged 15 to 44 to allow and promote research on male fertility and fatherhood.^1^

Until 2002, the NSFG was fielded periodically at approximately five-year intervals. Starting in 2006, the survey moved to continuous data collection, with data made available in two-year and four-year releases. Given our focus on recent trends, we draw on data collected between 2011 and 2019.^2^ The samples and sampling weights are designed to provide estimates for two-year periods centered at the mid-point, i.e., 2012, 2014, 2016, and 2018. Starting in 2016, the NSFG expanded the age range for both men and women to respondents aged 15 to 49; to assure comparability across years, we restrict our analyses to men and women aged 15 to 44 and exclude respondents with missing values on age or parity (21 respondents). This results in a sample of 17,928 men and 21,582 women. Table 1 reports additional sample size information by age and parity among all individuals in the sample, those who intend to have a(nother) child, and those with births in the two years preceding the interview, three key groups in our analyses.

**Table 1:** Sample size by parity and age among all individuals in the sample, those who intend to have a(nother) child, and those with births in the two years preceding the interview

	Men				Women			
	2012	2014	2016	2018	2012	2014	2016	2018
*Intentions to have a(nother) child*								
Aggregate	4,174	3,892	3,489	4,062	4,455	4,597	4,020	4,434
Parity								
0	2,790	2,579	2,405	2,793	2,252	2,396	2,091	2,481
1	611	575	480	542	956	928	818	803
2	449	487	375	442	753	766	693	664
3 or more	324	251	229	285	494	507	418	486
Age								
15–24	1,845	1,677	1,478	1,642	1,947	1,919	1,631	1,756
25–34	1,441	1,361	1,220	1,458	1,673	1,730	1,543	1,759
35–44	888	854	791	962	835	948	846	919
*Timing of positive fertility intentions*								
Aggregate	3,063	2,795	2,538	2,901	2,926	2,933	2,501	2,698
Parity								
0	2,391	2,188	1,986	2,286	1,841	1,927	1,621	1,883
1	395	362	320	343	633	587	499	463
2	177	175	160	193	306	270	274	229
3 or more	100	70	72	79	146	149	107	123
Age								
15–24	1,674	1,504	1322	1,428	1,650	1,626	1,323	1,429
25–34	1,071	967	883	1,085	1,050	1,053	959	1,026
35–44	318	324	333	388	226	254	219	243
*Retrospective fertility wantedness*								
Aggregate	466	439	359	400	753	739	676	601
Birth Order								
1	195	195	145	160	293	270	225	190
2	138	149	111	131	230	229	247	203
≥ 3	133	95	103	109	230	240	204	208
Age								
15–24	91	68	54	47	248	206	169	136
25–34	271	258	209	234	404	409	399	368
35–44	104	113	96	119	101	124	108	97

NSFG data from 2011 to 2019, men and women between age 15 and 44

We compute age- and parity-specific estimates of (i) the proportion of positive prospective fertility intentions, (ii) the timing of prospective fertility intentions, and (iii) the retrospective reporting of fertility desires.

In the NSFG, fertility goals questions are asked following a stepwise design. First, respondents are asked whether they *desire* to have a(nother) child in the future, as “(Looking to the future, do/If it were possible, would) you, yourself, want to have (a/nother) baby at some time (after this pregnancy is over/in the future)?”, with available answers “yes”, “no”, and “don’t know.” Second, respondents are asked whether they *intend* to have a(nother) child in the future, as “Do you (and [name of current married or cohabiting partner]) intend to have (a/nother) baby at some time (after this pregnancy is over/in the future)?”, with available answers “yes”, “no”, and “don’t know.” Respondents who do not desire another child and who are not currently married or cohabiting with a different-sex partner are not asked about their fertility intentions, as it is assumed they do not intend another child, and are accordingly coded as “no.” Respondents who are not physically able to have children (according to the NSFG, either surgically or nonsurgically sterile), or whose partners are not able, are asked about their fertility desires but not about their fertility intentions and are thus excluded from our main analyses (6,364 respondents, about 16% of the sample).

In the main analyses, we focus on fertility intentions rather than desires because (i) they imply actual plans and are thus more sensitive to external circumstances and time trends and (ii) we also have information on the timing of prospective fertility intentions, which provides a full picture of the quantum and timing of fertility intentions. In exploratory analyses we looked at fertility desires and found similar trends and patterns; figures are available on request. As supplementary analyses, recognizing that sterility status plays an important role in the measurement of fertility intentions (Badolato, 2025), we include in the Online Supplement an alternative specification of fertility intentions that includes individuals who are not (or whose partners are not) physically able to have children as a separate category of fertility intentions (see Online Supplement Figure S1). If the proportion of individuals who are not physically able to have children is changing across the study period (e.g., an increase in the proportion of individuals who opt-in to permanent contraception because they do not want and do not intend more children), this could affect trends in the proportion of individuals who intend more children. These additional analyses account for this possibility.

We define the proportion of positive prospective fertility intentions as the proportion of individuals who intend a(nother) child in the future. Measures of the timing of prospective fertility intentions are defined based on a direct question. The NSFG asks respondents who intend more children “When do you (and [name of current married or cohabiting partner]) expect your (next/first) child to be born (after this pregnancy)? Would you say, within the next two years, two–five years from now, or more than five years from now?” We include “don’t know” answers (176 respondents, 0.8% of the sample) in the “more than five years” category.

Finally, we define the retrospective reporting of fertility desires for births that occurred within the two years prior to the interview as the proportion of respondents who classified their last birth as occurring when no more pregnancies were wanted (“unwanted”), later than wanted, sooner than wanted, at the right time, or “doesn’t know or did not care.” For women, this classification is available as a recoded variable in the pregnancy file and retrieved from a series of questions about each pregnancy, while for men it is available as a recoded variable for each biological child. The question wording is similar for men and for women.

For each indicator, we first report aggregate-level estimators. We then divide respondents into three age groups (with age measured at the time of interview) and report age-specific estimators for ages 15–24, 25–34, and 35–44. Finally, we report parity-specific estimators for prospective fertility intentions and birth-order-specific estimators for the retrospective reporting of fertility desires. For each indicator, we report trends by computing the difference between 2012 and 2018 and present statistical t-tests for differences in proportions (for a comparable analysis using the NSFG data, see Hartnett & Gemmill, 2020). As an additional statistical test for time trends, we also compute chi-square test statistics for changes in the distribution of responses, which show robust results (not shown). For the two estimators based on prospective fertility intentions, we added one child to current parity, as recoded in the NSFG, if respondents or their partners were currently pregnant, since fertility intentions questions refer to the period after the pregnancy is over. For the estimator based on retrospective fertility desires, we include in the analyses respondents who had a live birth in the two years preceding interview, based on the age of the youngest child, which is computed by the NSFG from the date of birth. (Because the public-release dataset no longer contains the month of birth, we are not able to calculate directly from the birth histories.)

For women, information on live births, like information on retrospective reporting of fertility desires, is available in the pregnancy file. All live births are included in the analyses. For men, in order to minimize non-reporting bias in birth histories, the NSFG asks questions for biological children with a series of different types of partners (current wives or cohabiting partners, recent sexual partners, former wives and cohabiting partners, other sexual partners) and other children ever fathered or adopted. Despite these efforts, men’s fertility is likely underreported in the NSFG (Joyner et al., 2012); we return to this issue in the limitations section. We stratify our estimates by birth order of the reference birth, adjusting for multiple births (72 twins and 1 triplet in the sample).^3^.

## Results

### Prospective Fertility Intentions

**Fig. 1:**
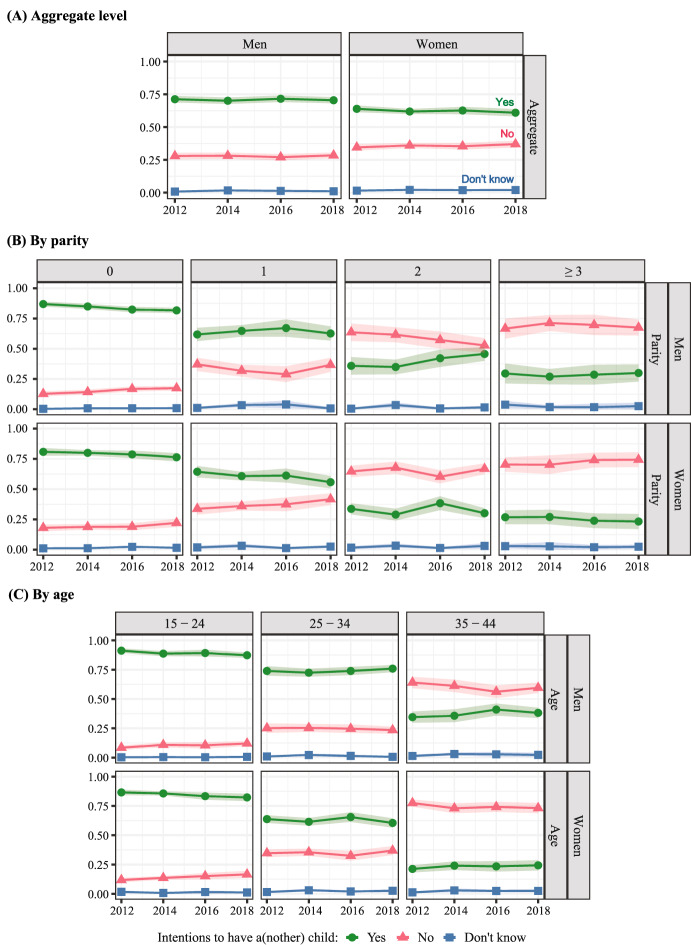
Trends in intentions to have a(nother) child at the aggregate level (panel a), by parity (panel b), and by age (panel c), weighted proportions and 95% Confidence Intervals. *Note*. NSFG data from 2011 to 2019; men and women between age 15 and 44 who are physically able (and, if partnered, whose partners are able) to have children. N = 33,123

Figure 1 shows intentions to have a(nother) child, separately for men and women. The top panel shows aggregate intentions; the second panel shows intentions by parity; and the third panel shows intentions by age. The green lines (circle markers) represent those who intend a(nother) child, the red (triangle markers) those who do not intend, and the blue (square markers) those who give “don’t know” responses. The shaded area around estimates shows 95% confidence intervals. Estimates, 95% confidence intervals, and statistical significance for trends for this and subsequent analyses are reported in tabular form in the Online Supplement (Online Supplement Tables S1, S2, S3, and S4). This figure addresses RQ1 and RQ2. Given the questions driving our analysis, we focus on trends over time and differences between men and women *within* parity and age categories, rather than differences *across* parity and age.

There are three main conclusions to be taken from Fig. 1. First, at the aggregate level, intentions to have another child declined over time for both men and women, with women reporting a greater decline. Second, these declines are concentrated early in the life course, especially for men. Trends at higher parities and older ages are different for men and women. Third, intentions to have another child are higher for men than for women in all age and parity groups as well as at the aggregate level. In particular, men report higher positive intentions at older ages than women, suggesting gendered life course trajectories in the timing of childbearing. Below, we expand on these conclusions.

From 2012 to 2018, aggregate-level intentions to have a(nother) child declined from 71 to 70% for men and from 64 to 61% for women. These declines are statistically significant, although relatively small in magnitude, representing a 1.4% decline from the starting value for men and a 4.7% decline for women.

The decline in positive intentions, and the corresponding increase in negative intentions, is especially marked at parity zero. In 2012, 87% of men and 81% of women without children intended to have a child, while in 2018 the proportions decreased to 82% of men and 76% of women. For both men and women, these numbers represent around a 6% decline from starting levels in the proportion of childless people intending a child. Similarly, intentions to have a child in the youngest age group (age 15–24) significantly decreased over the period of study for both men and women, suggesting potential cohort changes with successive cohorts of young adults less likely to intend children. In 2012, 91% of men and 87% of women age 15–24 intended to have a(nother) child, while in 2018 87% of men and 82% of women held these positive intentions.

For women, the proportion intending to have a(nother) child also declined significantly at parities one and two and at ages 25–34. For men, changes in positive intentions at higher parities and older ages were positive in direction but not statistically significant. Thus, there is some suggestion of diverging trends for men and women at higher parities and in the older age group.

A larger proportion of men than women have positive fertility intentions, and a smaller proportion have negative intentions. In 2018, at the aggregate level, 70% of men and 61% of women intended to have (more) children. Men and women were about equally likely to give “don’t know” responses, with estimates ranging from 1 to 2% across the study period. Consistent with the aggregate figure, positive intentions are higher for men than for women at all parities. As expected, positive intentions decline with parity for both men and women. (It is important to note that substantial proportions of people at high parities report that they or their partner are physically unable to have children; population proportions of intending another child are substantially lower when these people are included in the denominator, as shown in the Online Supplement Figure S1).

For both men and women, intentions to have a(nother) child are highest early in the life course and lowest toward the end of the reproductive years. Differences between men and women in the proportion of positive intentions increase across age groups, suggesting gendered life course trajectories. While the difference in positive intentions is small in the 15–24 age group, it is larger in the 35–44 age group. For example, in 2018, 38% of men age 35–44 intended to have more children, in contrast to 24% of women. Again, overall proportions are highly sensitive to the inclusion of people physically unable to have children, while time trends are robust (see Online Supplement Figure S1).

### Timing of Prospective Fertility Intentions

**Fig. 2:**
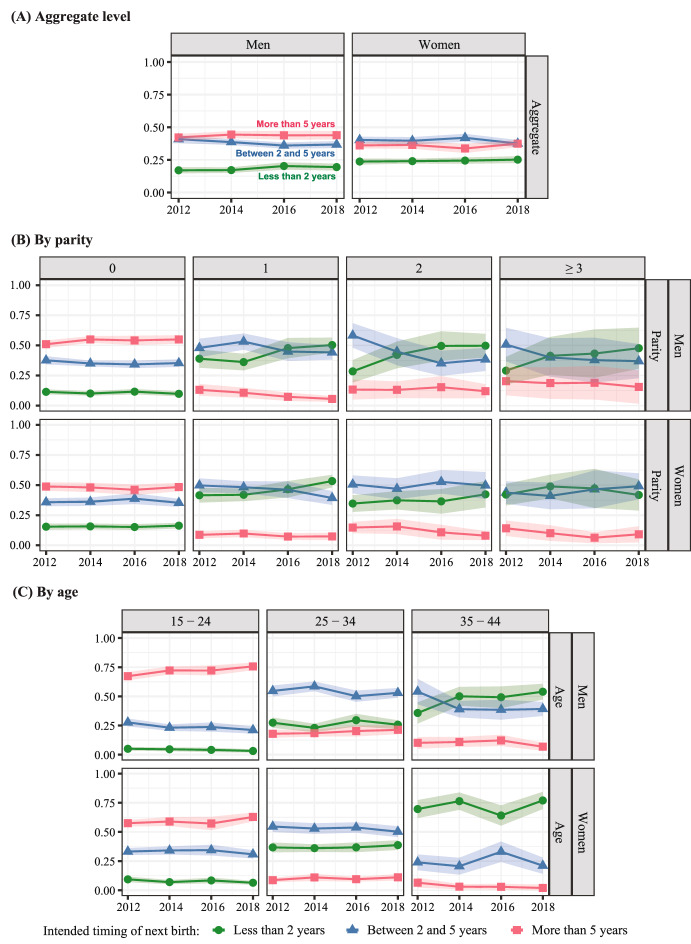
Trends in the intended timing of next birth at the aggregate level (panel a), by parity (panel b), and by age (panel c), weighted proportions and 95% Confidence Intervals. *Note* NSFG data from 2011 to 2019; men and women between age 15 and 44 who intend to have more children. N = 22,355

Figure 2 focuses on the intended timing of the next pregnancy for those respondents who say they intend to have a(nother) child. As with Fig. 1, the top panel shows aggregate proportions, the second panel disaggregates by parity, and the third panel disaggregates by age. The green line (circle markers) represents those who intend a child in less than two years, the blue line (triangle markers) between two and five years, and the red line (square markers) in more than five years or don’t know. The colored area around estimates shows 95% confidence intervals. This figure addresses RQ3.

We draw three main conclusions from Fig. 2. First, as with Fig. 1, change over time in the timing of prospective fertility intentions is small at the aggregate level. Second, men show later intended fertility than women. On aggregate, among those who intend another child, more men than women intend to wait for at least two years, and this difference is more marked at older age groups. Together with the results about the proportions of fertility intentions presented in the previous section, this result confirms gendered life course trajectories in the timing of childbearing. Third, among young men and women in the 15–24 age group, there is a significant increase over time in intentions to wait more than five years, with men reporting a greater increase.

Overall, there is little time trend in the intended timing of fertility for either men or women when looking at the aggregated figures. The only statistically significant trends are an increase of two percentage points in the proportion of men who intend to have a child in less than two years and a decline of three percentage points in the proportion of women who intend to wait between two and five years.

The second panel shows timing intentions disaggregated by parity. Consistent with aggregate intentions, there is little time trend at parity zero. However, there is a statistically significant increase in the proportion of men and women at parity one who intend to have a second child in less than two years. From 2012 to 2018, this proportion increased by 11 points for men and 12 points for women. This increase is consistent with the postponement of first births and suggests intentions to recuperate faster at older ages or, possibly, mismatches between timing intentions and realizations at parity one. There is some fluctuation in the figures for higher parities, but the sample sizes here are relatively small, as reflected in the wide confidence intervals, and the fluctuations may be due to sampling variation.

The third panel shows timing intentions disaggregated by age. In the youngest age group, age 15–24, there is a clear and statistically significant upward trend in intending to wait at least five years. In 2012, 67% of men and 57% of women in the 15–24 age group intended to wait at least five years, while in 2018 the proportions increased to 76% of men and 63% of women. This represents a 12.5% increase from the starting value for men and a 9.3% increase for women. This upward trend is weaker and not statistically significant, but still visible, among those age 25–34. Together, these patterns indicate continued postponement of childbearing in the United States.

The proportion of people intending a child soon increases with age, especially for women, reflecting both biological limitations to childbearing at older ages and social norms about appropriate ages for parenthood. In all age groups, women are more likely to intend a child soon than men, with gender differences particularly striking at older ages. In 2018, 77% of women age 35–44 who intended a child intended to have it within two years, in contrast to only 54% of men.

### Retrospective Reporting of Fertility Desires

**Fig. 3:**
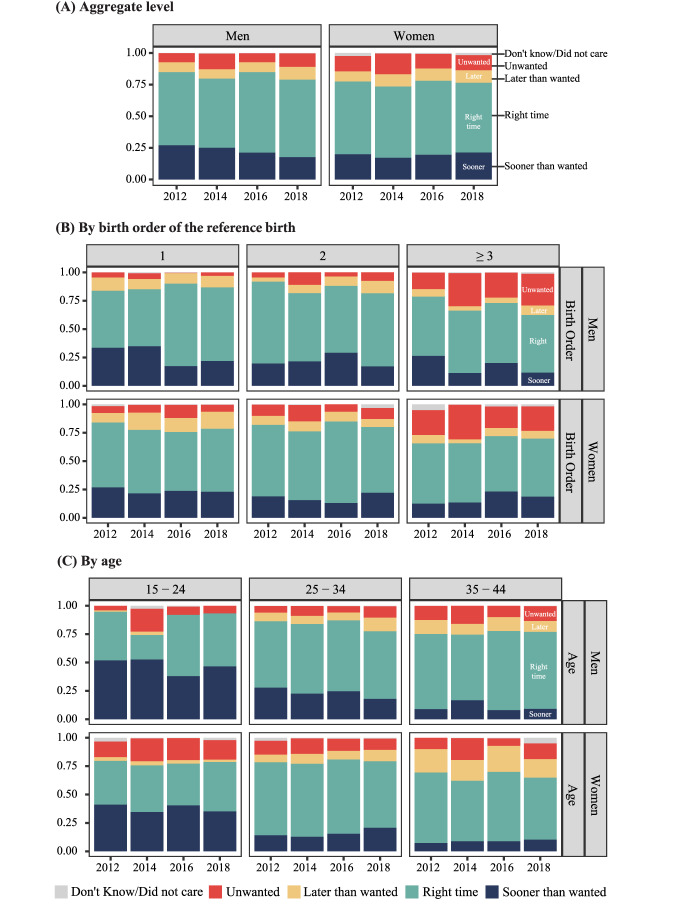
Trends in retrospective fertility wantedness at the aggregate level (panel a), by birth order of the reference birth (panel b), and by age (panel c), weighted proportions (95% Confidence Intervals available in Online Supplement Table S3). *Note*. NSFG data from 2011 to 2019; men and women between age 15 and 44 who had a child in 2 years before the interview. N = 4,433

Figure 3 presents the retrospective reporting of wantedness at the time of conception for children born in the two years prior to the survey, describing pregnancies as occurring sooner than wanted, at the right time, later than wanted, unwanted, and those for which the respondent didn’t know or didn’t care about wantedness. The three panels show aggregate-level wantedness and births disaggregated by birth order and age (at the time of the survey). This figure addresses RQ3.

Overall, the largest proportion of births were reported to have occurred at the right time for both men and women. In 2018, 61% of men and 55% of women reported their previous births occurring at the right time. At the aggregate level, men are slightly more likely than women to say that births took place earlier than desired, and women are slightly more likely to report births as unwanted. Women are more likely to report births occurring later than desired than men, especially among older age groups.

There is no clear time trend in the distribution of births by wantedness at the aggregate level. The only statistically significant trend is a decline in the proportion of men reporting births occurring sooner than wanted from 27% in 2012 to 18% in 2018. As a result, the proportion of men and women reporting births occurring sooner than wanted has become more similar across the study period, representing 18% of births in 2018 for men and 21% for women.

Differences across age and parity are consistent with previous research and are similar between men and women. The proportion of births occurring at the right time is higher for first and second births but lower for third and higher-order births, while the proportion reported as unwanted increases with parity. Among respondents age 15–24, there is a high proportion of births occurring sooner than wanted for both men and women, while among those age 25–34 and 35–44, the majority of births occurred at the right time. Among respondents age 35–44, a substantial proportion of births took place later than desired.

## Discussion and Conclusion

In this article, we described recent trends in the quantum and timing of fertility intentions together with trends in retrospectively reported wantedness of births, looking at all three elements of fertility goals together and looking at both men and women. This analysis moves beyond previous research to provide a fuller picture of changing fertility goals for both men and women in the United States and considers gender differences in both levels and trends. These analyses were motivated in part by a growing body of sociological and demographic research arguing for the inclusion of men in fertility research, as well as theoretical and empirical evidence suggesting that men and women might have different childbearing goals across the life course. Our analysis was guided by three specific research questions.

*Has the quantum of intended fertility declined over time for men and women, and if so, has it declined at the same rate for men and women? Do trends vary by age or parity? (RQ1)* On the aggregate, intentions to have a(nother) child declined between 2012 and 2018, consistent with trends reported by Hartnett and Gemmill (2020), and declined more for women than for men. Aggregate declines were statistically significant but relatively small (a decline of 4.7% relative to the starting value for women and 1.4% for men). Declines were proportionally larger early in the life course, i.e., for childless men and women and those age 15–24. For women, but not for men, intentions to have another child also declined significantly at higher parities and older ages.

*Are men more or less likely to intend to have no children than women, and is the prevalence of intended childlessness converging, diverging, or remaining stable between men and women? (RQ2)* Consistent with previous research showing gendered returns to childbearing and social expectations, men are less likely to intend childlessness than women. The prevalence of intended childlessness is increasing among both men and women, and as a result the gender gap in intended childlessness has remained largely stable over time.

*Do men have later desired and intended fertility than women, and do trends in the timing of desired and intended fertility differ for men and women? (RQ3)* Our results reveal gendered trajectories in the timing of childbearing. The gender gap in intentions to have a(nother) child is largest at older ages, and men are more likely to report intentions to delay childbearing than women, especially at older ages. Early in the life course, among the 15–24 age group, there is a significant increase in intentions to wait more than five years, with a greater increase among men (an increase of 9.3% relative to the starting value for women and 12.5% for men). These gender differences are reflected in the retrospective reporting of fertility desires as well. Women are more likely than men to retrospectively report a birth as unwanted or later than desired, especially among older respondents. In contrast, men are more likely than women to report a birth as earlier than desired, especially among younger respondents. However, in the aggregate, the proportion of men reporting a birth earlier than desired significantly declined, making gender differences in such births more similar across the study period.

In broad strokes, our results show strong similarities in the quantum of fertility goals between men and women. Across the period of study, on the order of four in five childless people intend to have a child, with this proportion declining over time for both men and women. For both men and women, the intention to have a(nother) child declines with age and parity. The plurality of births are reported as occurring at the right time, and the proportion of births reported as unwanted – a “quantum failure” – increases with parity for both men and women.

Still, there are salient differences between men’s and women’s quantum goals. In aggregate and across almost all age and parity groups, women are less likely than men to intend a(nother) child, and intentions to have a(nother) child declined more for women than for men. These differences are small in magnitude – on the order of five to six percentage points in most age and parity groups – but consistent.

As noted above in our summary of findings for RQ3, there are also consistent and meaningful differences in intended fertility timing for men and women, with men more likely to intend children at older ages than women and men more likely to intend to delay childbearing than women. Consistent with Kost et al. (2023), the substantial minority of women who reported that their first births took place later than desired suggests that timing intentions and timing desires may not be fully aligned, perhaps because life course conditions make it difficult for people to have children at the ages they would prefer. The significant decline for men, but not women, in the proportion of births occurring sooner than desired may suggest that, as a result of postponed fertility, men are more likely to have children at a time more consistent with their goals. Although couple-level age differences have narrowed over recent decades, men continue to be older than their female partners, and couples with men older than women are more common than the reverse (Lamidi et al., 2015). Men’s greater intentions to delay childbearing may reflect this age gap. Our results also align with prior research documenting gender differences in the ideal age at parenthood and perceived upper age limits for parenthood across European countries (Billari et al., 2021; Lazzari et al., 2024).

Although the shrinking gender differences in the division of labor and childcare might suggest that fertility goals are also becoming more similar for men and women, gender differences in the quantum and timing of fertility intentions at the aggregate level persist over time, with little evidence of either convergence or divergence. However, findings across parity and age groups reveal more complex trends. Among women, but not men, there is an increase in the proportion of negative intentions at parity one and two and among those aged 25–34, suggesting *growing* gender differences in the quantum of fertility intentions in these life stages. In contrast, among men, but not women, there is an increase in intentions to have the next child in less than two years at parities two and three or more, and men show a larger increase (compared to women) among those aged 35–44. Since men were less likely in 2012 to intend to have the next child in less than two years, this increase suggests *shrinking* gender differences in the timing of fertility intentions in these life stages. The decline in the proportion of men, but not women, reporting births occurring earlier than desired at the aggregate level also suggests *shrinking* gender differences in retrospective fertility wantedness. Our findings thus paint a mixed picture regarding gender convergence or divergence in fertility goals, depending on the exact outcome and life course stage being examined.

The impact of gender differences in fertility goals on future fertility behavior is unclear. In interpreting the gender differences shown in this article, it is important to note that the NSFG interviews independent samples of men and women. Some of the men and women in these samples are in coresidential partnerships, and questions about fertility goals in the NSFG refer to plans within these partnerships. For unpartnered men and women, fertility goals questions refer to individual plans; respondents may take hypothetical future partnerships into account when answering these questions. Thus, population-level differences between men and women may or may not translate into couple-level disagreements. Previous research shows that couples who disagree about fertility intentions are less likely to intend and have more children, especially at higher parities (Testa & Bolano, 2021; Testa et al., 2014). Similarly, couples who disagree about the timing of fertility intentions might decide to postpone childbearing, although strong norms of birth spacing persist in the United States (Guzzo, 2017).

The implications of gender similarities in fertility goals for future fertility behavior are more easily interpretable than the implications of gender differences. For instance, intended childlessness is increasing for both men and women. This trend suggests that voluntary childlessness may become more prevalent in the future, especially among recent cohorts. In addition to voluntary childlessness, delayed and postponed fertility is also a contributor to low period fertility. Current explanations of fertility postponement primarily focus on increasing female educational attainment and labor force participation and the incompatibility of work-family balance for working women (Mills et al., 2011). Although these are important mechanisms, our findings also show that men are more likely to intend to postpone childbearing than women, especially among older age groups. Incorporating men in these explanations would provide important, often overlooked perspectives.

### Limitations and Challenges in Male Fertility Research

Fathers’ underreporting of previous births has been extensively scrutinized (Bogges et al., 2006; Cherlin et al., 1983; Joyner et al., 2012; Martinez et al., 2006; Rendall et al., 1999). Joyner et al. (2012), comparing birth histories of men aged 15 to 24 in the NSFG 2002 with data from Vital Statistics and the U.S. Census, show that up to 20% of births in the population are not reported in the NSFG, with underestimation greatest for non-marital births. This underestimation is driven by both individual-level underreporting and exclusions from the sample frame. For instance, the NSFG excludes institutionalized respondents, such as those in prisons and in the military, who are more likely to be male.

It is important to note that not all estimators are equally affected by the underreporting of previous births. For aggregate and age-specific estimators of prospective fertility goals, no bias is introduced by the underreporting of previous births since those estimators do not depend on birth histories. For estimators of retrospective fertility desires only based on recent births, the underreporting of previous births is likely less than the 20% in full birth histories, although the exact degree of underreporting is unknown. Parity-specific indicators are more subject to the underreporting of previous births and, as underlined in the results section, interpreted with caution. Assuming that the bias introduced by underreporting of previous births is similar across the study period, time trend estimates should be relatively reliable.

In addition to these well-documented issues with reporting of men’s fertility, there are also concerns with data quality related to response rates. These concerns apply both to women and men. In the NSFG, as across most national surveys, response rates are declining, from 73.4% for women and 72.1% for men in the 2011–2013 release to 65.2% for women and 61.4% for men in the 2017–2019 release (2017–2019 NSFG summary of design and data collection methods).

In addition to the potential biases introduced by the underreporting of previous births and sampling frames, we want to highlight an additional set of constraints that limit the use of male fertility data. In the NSFG, men are asked different and fewer questions than women. For example, in addition to their own prospective and retrospective fertility desires, women are asked about the prospective and retrospective fertility desires of their partners, while men are not, as if partners’ fertility goals were less relevant for men than for women. Furthermore, non-partnered men are not asked about the certainty of their positive fertility intentions (how sure they are they will realize their intentions to have more children), as if they could not have children in the near future, while non-partnered women are asked about it. These limitations, in addition to the NSFG definition of partnered respondents as those married or cohabiting with a different-sex partner and the limited information on respondents’ gender identity, reflect a broader heteropatriarchal influence on the way standard demographic surveys collect fertility data. The NSFG pioneered the inclusion and promotion of men in fertility research and is one of the few national fertility surveys to include male respondents, providing much-needed insights on male fertility. Highlighting and addressing this set of overlooked concerns might further promote male fertility research.

### Conclusion

In contexts of declining and postponed fertility, understanding and predicting population-level fertility trends require a focus on childbearing goals and attitudes across age groups and parity levels and for both men and women. To date, declines in period fertility are driven in large part by declines in birth rates for younger age groups, and completed cohort fertility has not yet declined appreciably (Wu & Mark, 2023). It is not clear whether ongoing declines in early childbearing will be translated into lower completed fertility for cohorts still in their childbearing years. Our analysis showed increases in intended childlessness as well as increased intentions for delay among young people who intend children, both for men and for women. These two processes together point to potential future declines in cohort fertility, both through unrealized fertility and through voluntary childlessness. As underlined by Berrington (2004), there is a substantial group of “perpetual postponers” who want to have children early in the life course but, after some years of postponement, eventually decide not to have children. We show that men are more likely than women to intend children when surveyed early in the life course, but also more likely than women to intend to delay childbearing. These findings suggest potentially high levels of unrealized fertility goals for men as well as women, but little existing research has considered the impact of postponed or unrealized fertility for men.

Our findings suggest areas for future research that should critically include men’s fertility goals. Understanding the factors that influence men to postpone fertility goals would bring new insights into the process and mechanisms of fertility postponement, both by shedding light on potential disagreement at the couple level and by introducing new potential factors for understanding postponement. Long work hours, limited availability of paternity leave, perceived and observed workplace discrimination for fathers who take parental leave, and a broader culture that emphasizes the importance of male careers might be important drivers of fertility postponement among men (Billingsley & Ferrarini, 2014; Cha, 2010). Similarly, trends in the division of labor, parenting ideals, and gender inequality in labor force participation, key dimensions for fertility decision-making, might be important drivers for men’s and women’s fertility goals. Studying the ways that social structures shape childbearing goals and behaviors for men as well as for women is important to fully understand contemporary low-fertility regimes.

## Supplementary Information

Below is the link to the electronic supplementary material.

Supplementary file1 (DOCX 488 KB)

## Data Availability

National Survey of Family Growth data used for this study are publicly available and can be downloaded from the following link: https://www.cdc.gov/nchs/nsfg/index.htm.

## Footnotes

1 The NSFG releases female and male data in two separate files without further measures or specifications of gender identity.

2 The measure on the timing of prospective fertility intentions was added starting with the 2011–2013 data release, providing another reason to focus on this recent period.

3 For women, information on multiple births is available in the pregnancy file: for each pregnancy, women are asked the number of babies born alive from that pregnancy. For men, the information is not directly recoded by the NSFG as other information about biological children, but is available in each section about biological children with current wives or cohabiting partners, recent sexual partners, former wives and cohabiting partners, and other biological children with other sexual partners: if the same date of birth is reported for two or more children, a follow-up question about whether it was a multiple birth is asked.
