# Supplementary material for: Trends and Levels in Men’s and Women’s Fertility Goals in the United States
Source: Popul Res Policy Rev. 2026 Jan 22;45(1):6. doi: 10.1007/s11113-025-09989-5 (PMC12827432; doi:10.1007/s11113-025-09989-5)
Supplement: Supplementary file 1 — Supplementary file1 (DOCX 488 KB) [file 11113_2025_9989_MOESM1_ESM.docx]

# **ONLINE SUPPLEMENT**

**Trends and Levels in Men’s and Women’s Fertility Goals in the United States**

The Online Supplement includes the following figures and tables:

- **Figure S1:** Trends in fertility intentions including individuals who are not physically able (or, if partnered, whose partners are not able) to have children at the aggregate level (panel a), by parity (panel b), and by age (panel c), weighted proportions and 95% Confidence Intervals.
- **Table S1:** Trends in intentions to have a(nother) child at the aggregate level, by parity, and by age, weighted proportions and 95% Confidence Intervals.
- **Table S2:** Trends in the timing of fertility intentions at the aggregate level, by parity, and by age, weighted proportions and 95% Confidence Intervals.
- **Table S3:** Trends in retrospective fertility wantedness at the aggregate level, by birth order of the reference birth, and by age, weighted proportions and 95% Confidence Intervals.
- **Table S4:** Trends in intentions to have a(nother) child including individuals who are not physically able (or, if partnered, whose partners are not able) to have children at the aggregate level, by parity, and by age, weighted proportions and 95% Confidence Intervals.

**Figure S1:** Trends in fertility intentions including individuals who are not physically able (or, if partnered, whose partners are not able) to have children at the aggregate level (panel a), by parity (panel b), and by age (panel c), weighted proportions and 95% Confidence Intervals.

| **(A) Aggregate level**   |
| --- |
| **(B) By parity**   |
| **(C) By age**   |
|  |

Note. NSFG data from 2011 to 2019; men and women between age 15 and 44. N = 39,487.

**Table S1:** Trends in intentions to have a(nother) child at the aggregate level, by parity, and by age, weighted proportions and 95% Confidence Intervals.

|  |  | **Men** | | | **Women** | | |
| --- | --- | --- | --- | --- | --- | --- | --- |
| **Year** | **Level** | **Yes** | **No** | **Don’t know** | **Yes** | **No** | **Don’t know** |
| 2012 | Aggregate | .71 (.69 – .74) | .28 (.25 – .30) | .01 (.00 – .01) | .64 (.61 – .67) | .35 (.32 – .37) | .02 (.01 – .02) |
| 2014 | Aggregate | .70 (.68 – .73) | .28 (.26 – .31) | .02 (.01 – .02) | .62 (.60 – .64) | .36 (.34 – .38) | .02 (.01 – .03) |
| 2016 | Aggregate | .72 (.69 – .74) | .27 (.25 – .29) | .01 (.01 – .02) | .63 (.60 – .65) | .35 (.33 – .38) | .02 (.01 – .03) |
| 2018 | Aggregate | .70 (.68 – .73) | .28 (.26 – .31) | .01 (.00 – .02) | .61 (.58 – .64) | .37 (.34 – .40) | .02 (.01 – .03) |
| Δ (2018–2012) | Aggregate | -0.01* | 0.01 | 0.00 | -0.03* | 0.02* | 0.00* |
| 2012 | Parity 0 | .87 (.85 – .89) | .13 (.11 – .15) | .00 (.00 – .01) | .81 (.78 – .84) | .18 (.15 – .21) | .01 (.00 – .02) |
| 2014 | Parity 0 | .85 (.83 – .87) | .14 (.12 – .16) | .01 (.00 – .01) | .80 (.78 – .82) | .19 (.17 – .21) | .01 (.01 – .02) |
| 2016 | Parity 0 | .82 (.80 – .85) | .17 (.15 – .19) | .01 (.00 – .02) | .79 (.76 – .82) | .19 (.16 – .22) | .02 (.01 – .03) |
| 2018 | Parity 0 | .82 (.80 – .84) | .17 (.15 – .19) | .01 (.00 – .02) | .76 (.73 – .80) | .22 (.19 – .25) | .02 (.01 – .02) |
| Δ (2018–2012) | Parity 0 | -0.05* | 0.05* | 0.01* | -0.04* | 0.04* | 0.00* |
| 2012 | Parity 1 | .62 (.56 – .67) | .37 (.32 – .43) | .01 (.00 – .02) | .64 (.60 – .69) | .34 (.29 – .39) | .02 (.01 – .03) |
| 2014 | Parity 1 | .65 (.60 – .70) | .32 (.27 – .37) | .03 (.02 – .05) | .61 (.57 – .64) | .36 (.32 – .40) | .03 (.01 – .05) |
| 2016 | Parity 1 | .67 (.60 – .74) | .29 (.22 – .36) | .04 (.01 – .07) | .61 (.55 – .67) | .37 (.32 – .43) | .01 (.00 – .02) |
| 2018 | Parity 1 | .63 (.57 – .68) | .37 (.31 – .43) | .01 (.00 – .02) | .56 (.51 – .61) | .42 (.37 – .47) | .03 (.01 – .04) |
| Δ (2018–2012) | Parity 1 | 0.01 | 0.00 | 0.00 | -0.09* | 0.08* | 0.01 |
| 2012 | Parity 2 | .36 (.29 – .43) | .64 (.56 – .71) | .01 (.00 – .01) | .34 (.29 – .38) | .65 (.60 – .69) | .02 (.00 – .03) |
| 2014 | Parity 2 | .35 (.29 – .41) | .62 (.55 – .68) | .04 (.01 – .06) | .29 (.24 – .34) | .68 (.63 – .73) | .03 (.01 – .05) |
| 2016 | Parity 2 | .42 (.35 – .49) | .57 (.50 – .64) | .01 (.00 – .01) | .38 (.33 – .44) | .60 (.55 –.66) | .01 (.01 – .02) |
| 2018 | Parity 2 | .46 (.40 – .51) | .53 (.47 – .59) | .02 (.00 – .03) | .30 (.26 – .34) | .67 (.62 – .71) | .03 (.01 – .05) |
| Δ (2018–2012) | Parity 2 | 0.10 | -0.11 | 0.01 | -0.04* | 0.02* | 0.01 |
| 2012 | Parity ≥ 3 | .30 (.21 – .38) | .67 (.58 – .75) | .04 (.01 – .07) | .27 (.21 – .33) | .70 (.64 – .76) | .03 (.01 – .05) |
| 2014 | Parity ≥ 3 | .27 (.21 – .33) | .71 (.65 – .78) | .02 (.00 – .03) | .27 (.21 – .33) | .70 (.63 – .78) | .03 (.00 – .06) |
| 2016 | Parity ≥ 3 | .29 (.20 – .37) | .70 (.61 – .78) | .02 (.00 – .05) | .24 (.18 – .30) | .74 (.68 –.80) | .02 (.00 – .04) |
| 2018 | Parity ≥ 3 | .30 (.23 – .37) | .68 (.61 – .74) | .03 (.00 – .05) | .23 (.17 – .30) | .74 (.68 – .80) | .02 (.01 – .04) |
| Δ (2018–2012) | Parity ≥ 3 | 0.00 | 0.01 | -0-01 | -0.04 | 0.04 | -0.01 |
| 2012 | Age 15–24 | .91 (.89 – .93) | .08 (.07 – .10) | .00 (.00 – .01) | .87 (.84 – .89) | .12 (.10 – .14) | .02 (.01 – .03) |
| 2014 | Age 15–24 | .89 (.87 – .91) | .11 (.09 – .13) | .00 (.00 – .01) | .86 (.84 – .87) | .14 (.12 – .15) | .01 (.00 – .01) |
| 2016 | Age 15–24 | .89 (.86 – .92) | .10 (.08 – .13) | .00 (.00 – .01) | .83 (.80 – .86) | .15 (.12 – .18) | .02 (.00 – .03) |
| 2018 | Age 15–24 | .87 (.85 – .90) | .12 (.10 – .14) | .01 (.00 – .01) | .82 (.79 – .85) | .17 (.14 – .20) | .01 (.00 – .02) |
| Δ (2018–2012) | Age 15–24 | -0.04* | 0.04* | 0.00 | -0.04* | 0.05* | -0.01 |
| 2012 | Age 25–34 | .74 (.70 – .78) | .25 (.21 – .29) | .01 (.00 – .02) | .64 (.60 – .67) | .35 (.31 – .38) | .02 (.01 – .02) |
| 2014 | Age 25–34 | .72 (.69 – .76) | .25 (.22 – .29) | .02 (.01 – .03) | .61 (.58 – .65) | .36 (.32 – .39) | .03 (.02 – .04) |
| 2016 | Age 25–34 | .74 (.70 – .77) | .25 (.21 – .28) | .01 (.00 – .03) | .66 (.61 – .70) | .32 (.28 – .37) | .02 (.01 – .03) |
| 2018 | Age 25–34 | .76 (.73 – .79) | .23 (.20 – .26) | .01 (.00 – .01) | .60 (.56 – .65) | .37 (.33 – .41) | .03 (.01 – .04) |
| Δ (2018–2012) | Age 25–34 | 0.02 | -0.02 | 0.00 | -0.03* | 0.02* | 0.01 |
| 2012 | Age 35–44 | .35 (.30 – .39) | .64 (.59 – .69) | .01 (.00 – .02) | .21 (.18 – .24) | .77 (.74 – .81) | .01 (.01 – .02) |
| 2014 | Age 35–44 | .36 (.30 – .41) | .61 (.56 – .67) | .03 (.02 – .04) | .24 (.20 – .28) | .73 (.69 – .77) | .03(.01 – .05) |
| 2016 | Age 35–44 | .41 (.36 – .46) | .56 (.51 – .62) | .03 (.01 – .05) | .23 (.19 – .28) | .74 (.70 – .78) | .02 (.01 – .04) |
| 2018 | Age 35–44 | .38 (.34 – .43) | .60 (.55 – .64) | .02 (.00 – .04) | .24 (.20 – .29) | .73 (.69 – .78) | .03 (.01 – .04) |
| Δ (2018–2012) | Age 35–44 | 0.04 | -0.05* | 0.01 | 0.03 | -0.04 | 0.01 |

Note. NSFG data from 2011 to 2019; men and women between age 15 and 44 who are physically able (and, if partnered, whose partners are able) to have children. N = 33,123.

* p < .05 Test of equal proportions in 2012 and 2018.

**Table S2:** Trends in the timing of fertility intentions at the aggregate level, by parity, and by age, weighted proportions and 95% Confidence Intervals.

|  |  | **Men** | | | **Women** | | |
| --- | --- | --- | --- | --- | --- | --- | --- |
| **Year** | **Level** | **< 2Y** | **2Y–5Y** | **>5Y** | **< 2Y** | **2Y–5Y** | **>5Y** |
| 2012 | Aggregate | .17 (.15 – .19) | .41 (.38 – .44) | .42 (.40 – .45) | .24 (.21 – .26) | .40 (.38 – .43) | .36 (.34 – .38) |
| 2014 | Aggregate | .17 (.15 – .19) | .39 (.36 – .41) | .44 (.42 – .47) | .24 (.22 – .26) | .40 (.37 – .42) | .36 (.33 – .39) |
| 2016 | Aggregate | .20 (.17 – .23) | .36 (.34 – .38) | .44 (.40 – .47) | .24 (.22 – .27) | .42 (.39 – .45) | .34 (.30 – .37) |
| 2018 | Aggregate | .19 (.17 – .22) | .37 (.34 – .39) | .44 (.41 – .47) | .25 (.23 – .28) | .37 (.35 – .40) | .37 (.35 – .40) |
| Δ (2018–2012) | Aggregate | 0.02* | -0.04 | 0.02 | 0.01 | -0.03* | 0.01 |
| 2012 | Parity 0 | .11 (.09 – .13) | .38 (.34 – .41) | .51 (.48 – .54) | .15 (.13 – .18) | .36 (.32 – .39) | .49 (.45 – .52) |
| 2014 | Parity 0 | .10 (.08 – .12) | .35 (.33 – .37) | .55 (.52 – .58) | .16 (.13 – .18) | .36 (.33 – .40) | .48 (.44 – .52) |
| 2016 | Parity 0 | .12 (.09 – .14) | .34 (.31 – .37) | .54 (.50 – .58) | .15 (.13 – .18) | .39 (.34 – .43) | .46 (.42 – .51) |
| 2018 | Parity 0 | .10 (.08 – .12) | .35 (.32 – .38) | .55 (.51 – .58) | .16 (.14 – .19) | .35 (.32 – .39) | .48 (.45 – .52) |
| Δ (2018–2012) | Parity 0 | -0.02 | -0.02 | 0.04 | 0.01 | 0.00 | 0.00 |
| 2012 | Parity 1 | .39 (.31 – .47) | .48 (.40 – .56) | .13 (.08 – .18) | .42 (.35 – .48) | .50 (.44 – .56) | .09 (.06 – .12) |
| 2014 | Parity 1 | .36 (.29 – .43) | .53 (.46 – .60) | .11 (.06 – .15) | .42 (.37 – .47) | .48 (.43 – .53) | .10 (.07 – .13) |
| 2016 | Parity 1 | .48 (.39 – .56) | .45 (.37 – .53) | .07 (.04 – .11) | .47 (.40 – .53) | .46 (.39 – .53) | .07 (.04 – .10) |
| 2018 | Parity 1 | .50 (.44 – .57) | .44 (.38 – .50) | .06 (.03 – .08) | .53 (.48 – .59) | .39 (.34 – .45) | .07 (.04 – .10) |
| Δ (2018–2012) | Parity 1 | 0.11* | -0.04 | -0.08* | 0.12* | -0.10 | -0.01 |
| 2012 | Parity 2 | .28 (.19 – .38) | .58 (.48 – .68) | .13 (.05 – .22) | .35 (.28 – .42) | .51 (.43 – .58) | .15 (.10 – .19) |
| 2014 | Parity 2 | .42 (.31 – .53) | .45 (.34 – .56) | .13 (.06 – .20) | .37 (.30 – .45) | .47 (.38 – .56) | .16 (.09 – .22) |
| 2016 | Parity 2 | .50 (.37 – .62) | .35 (.25 – .46) | .15 (.06 – .24) | .36 (.26 – .47) | .53 (.43 – .62) | .11 (.05 – .17) |
| 2018 | Parity 2 | .50 (.40 – .60) | .38 (.29 – .48) | .12 (.06 – .18) | .42 (.31 – .53) | .50 (.38 – .61) | .08 (.04 – .12) |
| Δ (2018–2012) | Parity 2 | 0.21* | -0.20 | -0.01 | 0.08 | -0.01 | -0.07* |
| 2012 | Parity ≥ 3 | .29 (.18 – .40) | .51 (.37 – .65) | .20 (.09 – .32) | .42 (.33 – .51) | .44 (.34 – .53) | .14 (.08 – .21) |
| 2014 | Parity ≥ 3 | .41 (.26 – .57) ^a^ | .40 (.24 – .56) ^a^ | .19 (.06 – .32) ^a^ | .49 (.39 – .59) | .41 (.30 – .52) | .10 (.04 – .17) |
| 2016 | Parity ≥ 3 | .43 (.23 – .63) ^a^ | .38 (.19 – .56) ^a^ | .19 (.05 – .33) ^a^ | .47 (.31 – .63) | .46 (.31 – .62) | .06 (.02 – .11) |
| 2018 | Parity ≥ 3 | .48 (.31 – .65)^a^ | .37 (.23 – .51) ^a^ | .15 (.02 – .29) ^a^ | .42 (.29 – .55) | .49 (.38 – .60) | .09 (.02 – .16) |
| Δ (2018–2012) | Parity ≥ 3 | 0.19* | -0.14 | -0.05 | 0.00 | 0.05 | -0.05 |
| 2012 | Age 15–24 | .05 (.03 – .07) | .28 (.24 – .31) | .67 (.64 – .71) | .09 (.07 – .12) | .33 (.30 – .36) | .57 (.55 – .60) |
| 2014 | Age 15–24 | .05 (.03 – .06) | .23 (.20 – .26) | .72 (.69 – .76) | .07 (.05 – .09) | .34 (.31 – .38) | .59 (.55 – .63) |
| 2016 | Age 15–24 | .04 (.02 – .06) | .24 (.20 – .28) | .72 (.68 – .76) | .08 (.06 – .11) | .34 (.30 – .39) | .57 (.51 – .63) |
| 2018 | Age 15–24 | .03 (.02 – .05) | .21 (.18 – .24) | .76 (.72 – .79) | .06 (.05 – .08) | .31 (.27 – .34) | .63 (.59 – .67) |
| Δ (2018–2012) | Age 15–24 | -0.02* | -0.07* | 0.08* | -0.03 | -0.02* | 0.05* |
| 2012 | Age 25–34 | .27 (.23 – .32) | .55 (.50 – .59) | .18 (.14 – .21) | .37 (.32 – .41) | .55 (.50 – .59) | .09 (.07 – .11) |
| 2014 | Age 25–34 | .23 (.19 – .27) | .59 (.54 – .63) | .18 (.16 – .21) | .36 (.33 – .39) | .53 (.48 – .57) | .11 (.08 – .14) |
| 2016 | Age 25–34 | .30 (.24 – .35) | .50 (.45 – .55) | .20 (.16 – .25) | .37 (.33 – .41) | .54 (.49 – .58) | .09 (.07 – .11) |
| 2018 | Age 25–34 | .26 (.22 – .30) | .53 (.49 – .57) | .21 (.18 – .25) | .39 (.35 – .43) | .50 (.46 – .55) | .11 (.08 – .14) |
| Δ (2018–2012) | Age 25–34 | -0.02 | -0.02 | 0.03 | 0.02 | -0.04 | 0.02 |
| 2012 | Age 35–44 | .36 (.27 – .45) | .54 (.43 – .65) | .10 (.05 – .15) | .70 (.62 – .77) | .24 (.17 – .31) | .06 (.02 – .11) |
| 2014 | Age 35–44 | .50 (.42 – .58) | .39 (.32 – .46) | .11 (.07 – .15) | .76 (.69 – .84) | .21 (.13 – .28) | .03 (.01 – .05) |
| 2016 | Age 35–44 | .49 (.40 – .59) | .39 (.30 – .47) | .12 (.07 – .17) | .64 (.55 – .73) | .33 (.24 – .42) | .03 (.00 – .05) |
| 2018 | Age 35–44 | .54 (.47 – .61) | .39 (.33 – .45) | .07 (.03 – .10) | .77 (.70 – .84) | .21 (.14 – .28) | .02 (.00 – .05) |
| Δ (2018–2012) | Age 35–44 | 0.18* | -0.15 | -0.03 | 0.07* | -0.03 | -0.05 |

Note. NSFG data from 2011 to 2019; men and women between age 15 and 44 who intend to have more children. N = 22,355.

^a^ Sample size less than 100 observations.

* p < .05 Test of equal proportions in 2012 and 2018.

**Table S3:** Trends in retrospective fertility wantedness at the aggregate level, by birth order of the reference birth, and by age, weighted proportions and 95% Confidence Intervals.

|  |  | **Men** | | | | | **Women** | | | | |
| --- | --- | --- | --- | --- | --- | --- | --- | --- | --- | --- | --- |
| **Year** | **Level** | **DK** | **Unwanted** | **Later than wanted** | **Right time** | **Sooner than wanted** | **DK** | **Unwanted** | **Later than wanted** | **Right time** | **Sooner than wanted** |
| 2012 | Aggregate | .00 (.00 – .00) | .07 (.04 – .11) | .08 (.04 – .11) | .58 (.51 – .65) | .27 (.20 – .34) | .02 (.00 – .05) | .12 (.09 – .15) | .08 (.05 – .11) | .57 (.52 – .63) | .20 (.16 – .24) |
| 2014 | Aggregate | .01 (.00 – .01) | .12 (.08 – .17) | .07 (.05 – .10) | .55 (.49 – .61) | .25 (.19 – .31) | .00 (.00 – .01) | .16 (.12 – .21) | .10 (.07 – .13) | .56 (.52 – .61) | .17 (.14 – .21) |
| 2016 | Aggregate | .00 (.00 – .01) | .07 (.03 – .11) | .08 (.04 – .12) | .64 (.56 – .71) | .21 (.16 – .26) | .01 (.00 – .02) | .12 (.08 – .16) | .10 (.06 – .13) | .59 (.52 – .65) | .20 (.15 – .24) |
| 2018 | Aggregate | .00 (.00 – .01) | .11 (.07 – .15) | .10 (.05 – .15) | .61 (.55 – .68) | .18 (.14 – .21) | .02 (.00 – .03) | .12 (.08 – .16) | .10 (.06 – .14) | .55 (.50 – .60) | .21 (.17 – .25) |
| Δ (2018–2012) | Aggregate | 0.00 | 0.03 | 0.02 | 0.03 | -0.09* | -0.01 | 0.00 | 0.02 | -0.02 | 0.01 |
| 2012 | Birth 1 | .00 (.00 – .00) | .04 (.01 – .07) | .12 (.05 – .19) | .50 (.39 – .61) | .33 (.24 – .43) | .02 (.00 – .03) | .06 (.03 – .09) | .08 (.05 – .12) | .57 (.49 – .66) | .27 (.20 – .33) |
| 2014 | Birth 1 | .01 (.00 – .03) | .05 (.00 – .10) | .09 (.04 – .14) | .50 (.40 – .60) | .35 (.24 – .46) | .00 (.00 – .01) | .07 (.04 – .10) | .15 (.08 – .22) | .56 (.48 – .64) | .22 (.15 – .28) |
| 2016 | Birth 1 | .00 (.00 – .01) | .00 (.00 – .01) | .09 (.04 – .15) | .73 (.63 – .83) | .17 (.10 – .25) | .01 (.00 – .01) | .11 (.03 – .20) | .13 (.07 – .18) | .52 (.41 – .63) | .24 (.16 – .31) |
| 2018 | Birth 1 | .00 (.00 – .00) | .03 (.00 – .06) | .10 (.03 – .17) | .65 (.54 – .76) | .22 (.15 – .29) | .00 (.00 – .01) | .06 (.00 – .12) | .15 (.09 – .21) | .56 (.47 – .64) | .23 (.15 – .31) |
| Δ (2018–2012) | Birth 1 | 0.00 | -0.01 | -0.02 | 0.15 | -0.12 | -0-01 | 0.00 | 0.07 | -0.02 | -0.04 |
| 2012 | Birth 2 | .00 (.00 – .00) | .04 (.01 – .08) | .04 (.00 – .07) | .72 (.62 – .82) | .20 (.10 – .29) | .00 (.00 – .01) | .10 (.05 – .15) | .08 (.04 – .12) | .63 (.55 – .71) | .19 (.13 – .25) |
| 2014 | Birth 2 | .00 (.00 – .00) | .11 (.06 – .16) | .07 (.03 – .11) | .60 (.50 – .70) | .22 (.12 – .31) | .01 (.00 – .01) | .14 (.06 – .22) | .09 (.04 – .14) | .61 (.51 – .70) | .16 (.10 – .21) |
| 2016 | Birth 2 | .00 (.00 – .00) | .04 (.01 – .06) | .08 (.02 – .15) | .59 (.46 – .72) | .29 (.18 – .40) | .00 (.00 – .00) | .06 (.03 – .09) | .09 (.04 – .13) | .72 (.64 – .79) | .13 (.08 – .18) |
| 2018 | Birth 2 | .00 (.00 – .00) | .07 (.03 – .12) | .11 (.03 – .19) | .64 (.54 – .75) | .17 (.11 – .24) | .03 (.00 – .08) | .10 (.05 – .15) | .07 (.04 – .11) | .58 (.49 – .67) | .22 (.13 – .31) |
| Δ (2018–2012) | Birth 2 | 0.00 | 0.03 | 0.07 | -0.08 | -0.02 | 0.03 | 0.00 | -0.01 | -0.05 | 0.03 |
| 2012 | Birth ≥ 3 | .00 (.00 – .01) | .14 (.05 – .24) | .07 (.01 – .13) | .52 (.41 – .64) | .26 (.14 – .38) | .05 (.00 – .12) | .22 (.14 – .29) | .07 (.01 – .14) | .53 (.43 – .63) | .13 (.07 – .18) |
| 2014 | Birth ≥ 3 | .01 (.00 – .02) | .29 (.16 – .42) | .04 (.00 – .08) | .55 (.42 – .68) | .11 (.03 – .19) | .00 (.00 – .01) | .30 (.23 – .38) | .04 (.01 – .06) | .52 (.44 – .61) | .13 (.08 – .19) |
| 2016 | Birth ≥ 3 | .00 (.00 – .01) | .22 (.09 – .35) | .05 (.01 – .09) | .53 (.40 – .65) | .20 (.08 – .32) | .02 (.00 – .06) | .19 (.12 – .26) | .07 (.01 – .13) | .49 (.38 – .59) | .23 (.11 – .35) |
| 2018 | Birth ≥ 3 | .01 (.00 – .03) | .28 (.16 – .40) | .08 (.00 – .17) | .51 (.38 – .63) | .12 (.05 – .18) | .02 (.00 – .04) | .22 (.13 – .30) | .07 (.02 – .12) | .51 (.42 – .60) | .19 (.11 – .26) |
| Δ (2018–2012) | Birth ≥ 3 | 0.01 | 0.13 | 0.02 | -0.01 | -0.15 | -0.03 | 0.00 | -0.01 | -0.02 | 0.06 |
| 2012 | Age 15–24 | .00 (.00 – .00) ^a^ | .04 (.00 – .08) ^a^ | .01 (.00 – .04) ^a^ | .43 (.31 – .55) ^a^ | .52 (.40 – .64) ^a^ | .03 (.00 – .07) | .14 (.08 – .19) | .03 (.00 – .07) | .39 (.30 – .47) | .41 (.33 – .49) |
| 2014 | Age 15–24 | .03 (.00 – .08) ^a^ | .20 (.05 – .35) ^a^ | .03 (.00 – .07) ^a^ | .22 (.10 – .33) ^a^ | .53 (.35 – .71) ^a^ | .01 (.00 – .01) | .20 (.11 – .29) | .04 (.00 – .08) | .41 (.32 – .50) | .35 (.25 – .44) |
| 2016 | Age 15–24 | .01 (.00 – .02) ^a^ | .07 (.00 – .19) ^a^ | .00 (.00 – .00) ^a^ | .54 (.25 – .83) ^a^ | .38 (.13 – .62) ^a^ | .00 (.00 – .01) | .19 (.08 – .30) | .03 (.00 – .07) | .37 (.26 – .48) | .40 (.30 – .51) |
| 2018 | Age 15–24 | .00 (.00 – .00) ^a^ | .07 (.00 – .15) ^a^ | .00 (.00 – .00) ^a^ | .47 (.24 – .70) ^a^ | .47 (.23 – .70) ^a^ | .02 (.00 – .05) ^a^ | .17 (.06 – .29) | .02 (.00 – .05) | .43 (.29 – .58) | .35 (.23 – .47) |
| Δ (2018–2012) | Age 15–24 | 0.00 | 0.03 | -0.01 | 0.04 | -0.05 | -0.01 | 0.03 | -0.01 | 0.05 | -0.06 |
| 2012 | Age 25–34 | .00 (.00 – .01) | .06 (.02 – .09) | .08 (.03 – .13) | .59 (.50 – .67) | .28 (.20 – .36) | .03 (.00 – .06) | .12 (.08 – .16) | .07 (.05 – .09) | .64 (.57 – .72) | .14 (.10 – .18) |
| 2014 | Age 25–34 | .00 (.00 – .01) | .09 (.05 – .13) | .07 (.04 – .11) | .61 (.53 – .69) | .23 (.15 – .30) | .01 (.00 – .01) | .14 (.09 – .18) | .09 (.05 – .13) | .64 (.58 – .70) | .13 (.09 – .16) |
| 2016 | Age 25–34 | .00 (.00 – .01) | .06 (.02 – .10) | .07 (.03 – .11) | .62 (.53 – .71) | .25 (.17 – .32) | .01 (.00 – .03) | .11 (.07 – .14) | .08 (.04 – .11) | .65 (.58 – .72) | .15 (.10 – .21) |
| 2018 | Age 25–34 | .00 (.00 – .01) | .10 (.05 – .15) | .12 (.04 – .20) | .60 (.51 – .68) | .18 (.12 – .24) | .01 (.00 – .01) | .10 (.06 – .13) | .10 (.06 – .15) | .59 (.51 – .66) | .21 (.15 – .27) |
| Δ (2018–2012) | Age 25–34 | 0.00 | 0.04 | 0.04 | 0.01 | -0.10 | -0.02 | -0.02 | 0.03 | -0.06 | 0.07 |
| 2012 | Age 35–44 | .00 (.00 – .01) | .12 (.04 – .20) | .12 (.05 – .20) | .66 (.54 – .78) | .09 (.03 – .15) | .00 (.00 – .00) | .10 (.03 – .17) | .21 (.08 – .34) | .62 (.48 – .76) | .07 (.02 – .13) |
| 2014 | Age 35–44 | .00 (.00 – .00) | .16 (.07 – .25) | .10 (.05 – .15) | .58 (.47 – .69) | .17 (.07 – .26) | .00 (.00 – .01) | .19 (.09 – .29) | .18 (.10 – .26) | .53 (.40 – .67) | .09 (.00 – .17) |
| 2016 | Age 35–44 | .00 (.00 – .00) ^a^ | .10 (.00 – .20) ^a^ | .12 (.04 – .21) ^a^ | .70 (.59 – .80) ^a^ | .08 (.02 – .15) ^a^ | .01 (.00 – .02) | .06 (.02 – .11) | .23 (.09 – .37) | .61 (.47 – .75) | .09 (.01 – .17) |
| 2018 | Age 35–44 | .00 (.00 – .01) | .13 (.05 – .21) | .10 (.02 – .17) | .68 (.58 – .77) | .09 (.05 – .14) | .05 (.00 – .13) ^a^ | .14 (.01 – .26) ^a^ | .16 (.07 – .26) ^a^ | .55 (.40 – .69) ^a^ | .10 (.03 – .18) ^a^ |
| Δ (2018–2012) | Age 35–44 | 0.00 | 0.01 | -0.03 | 0.02 | 0.00 | 0.05 | 0.04 | -0.04 | -0.07 | 0.03 |

Note. NSFG data from 2011 to 2019; men and women between age 15 and 44 who had a child in two years before the interview. N = 4,433.

^a^ Sample size less than 100 observations.

* p < .05 Test of equal proportions in 2012 and 2018.

**Table S4:** Trends in intentions to have a(nother) child including individuals who are not physically able (or, if partnered, whose partners are not able) to have children at the aggregate level, by parity, and by age, weighted proportions and 95% Confidence Intervals.

|  |  | **Men** | | | | **Women** | | | |
| --- | --- | --- | --- | --- | --- | --- | --- | --- | --- |
| **Year** | **Level** | **Yes** | **No** | **Don’t know** | **Sterile** | **Yes** | **No** | **Don’t know** | **Sterile** |
| 2012 | Aggregate | .59 (.57 – .62) | .23 (.21 – .25) | .01 (.00 – .01) | .17 (.15 – .19) | .49 (.47 – .52) | .26 (.25 – .28) | .01 (.01 – .02) | .23 (.21 – .25) |
| 2014 | Aggregate | .58 (.55 – .61) | .23 (.21 – .25) | .01 (.01 – .02) | .18 (.15 – .20) | .50 (.48 – .52) | .29 (.27 – .30) | .02 (.01 – .02) | .20 (.18 – .22) |
| 2016 | Aggregate | .60 (.57 – .63) | .23 (.21 – .25) | .01 (.01 – .02) | .16 (.14 – .19) | .50 (.47 – .52) | .28 (.26 – .30) | .02 (.01 – .02) | .20 (.18 – .23) |
| 2018 | Aggregate | .59 (.56 – .62) | .24 (.22 – .26) | .01 (.00 – .01) | .16 (.14 – .18) | .48 (.46 – .51) | .29 (.27 – .31) | .02 (.01 – .02) | .21 (.19 – .22) |
| Δ (2018–2012) | Aggregate | 0.00 | 0.01 | 0.00 | -0.01 | -0.01* | 0.03* | 0.00* | -0.03* |
| 2012 | Parity 0 | .82 (.80 – .84) | .12 (.10 – .14) | .00 (.00 – .01) | .06 (.04 – .07) | .78 (.74 – .81) | .17 (.15 – .20) | .01 (.00 – .02) | .04 (.03 – .05) |
| 2014 | Parity 0 | .79 (.77 – .82) | .13 (.11 – .15) | .01 (.00 – .01) | .06 (.05 – .08) | .76 (.74 – .79) | .18 (.16 – .20) | .01 (.01 – .02) | .04 (.03 – .06) |
| 2016 | Parity 0 | .77 (.74 – .79) | .16 (.14 – .18) | .01 (.00 – .01) | .07 (.05 – .09) | .75 (.72 – .78) | .18 (.15 – .21) | .02 (.01 – .03) | .05 (.03 – .06) |
| 2018 | Parity 0 | .76 (.73 – .78) | .16 (.14 – .18) | .01 (.00 – .01) | .07 (.06 – .09) | .73 (.69 – .76) | .21 (.18 – .24) | .01 (.01 – .02) | .05 (.04 – .06) |
| Δ (2018–2012) | Parity 0 | -0.06* | 0.04* | 0.01* | 0.02 | -0.05* | 0.04* | 0.00* | 0.01 |
| 2012 | Parity 1 | .52 (.47 – .57) | .31 (.26 – .37) | .01 (.00 – .02) | .15 (.11 – .20) | .56 (.52 – .61) | .30 (.25 – .34) | .02 (.01 – .03) | .13 (.10 – .15) |
| 2014 | Parity 1 | .54 (.49 – .60) | .27 (.22 – .31) | .03 (.01 – .04) | .16 (.12 – .20) | .55 (.52 – .59) | .33 (.29 – .36) | .03 (.01 – .04) | .09 (.07 – .11) |
| 2016 | Parity 1 | .55 (.48 – .63) | .24 (.18 – .29) | .03 (.01 – .06) | .18 (.12 – .24) | .53 (.47 – .59) | .32 (.27 – .37) | .01 (.00 – .02) | .14 (.10 – .18) |
| 2018 | Parity 1 | .54 (.48 – .59) | .31 (.26 – .37) | .01 (.00 – .01) | .14 (.11 – .18) | .50 (.45 – .54) | .37 (.32 – .42) | .02 (.01 – .03) | .11 (.09 – .14) |
| Δ (2018–2012) | Parity 1 | 0.01 | 0.00 | 0.00 | -0.01 | -0.07* | 0.07* | 0.01 | -0.01 |
| 2012 | Parity 2 | .24 (.18 – .30) | .42 (.37 – .48) | .00 (.00 – .01) | .34 (.27 – .40) | .21 (.17 – .24) | .39 (.35 – .44) | .01 (.00 – .02) | .39 (.34 – .44) |
| 2014 | Parity 2 | .23 (.19 – .27) | .40 (.34 – .47) | .02 (.01 – .04) | .35 (.28 – .41) | .19 (.16 – .23) | .45 (.41 – .49) | .02 (.01 – .03) | .34 (.30 – .37) |
| 2016 | Parity 2 | .30 (.25 – .35) | .41 (.34 – .47) | .00 (.00 – .01) | .29 (.23 – .34) | .26 (.22 – .30) | .40 (.36 – .45) | .01 (.00 – .01) | .33 (.28 – .37) |
| 2018 | Parity 2 | .31 (.27 – .36) | .36 (.31 – .41) | .01 (.00 – .02) | .31 (.26 – .36) | .18 (.15 – .21) | .40 (.36 – .44) | .02 (.00 – .03) | .40 (.36 – .45) |
| Δ (2018–2012) | Parity 2 | 0.08 | -0.06 | 0.01 | -0.02 | -0.03* | 0.00 | 0.01 | 0.01 |
| 2012 | Parity ≥ 3 | .16 (.11 – .22) | .37 (.31 – .43) | .02 (.00 – .04) | .44 (.37 – .52) | .11 (.09 – .14) | .30 (.26 – .34) | .01 (.00 – .02) | .58 (.53 – .63) |
| 2014 | Parity ≥ 3 | .14 (.10 – .19) | .38 (.32 – .44) | .01 (.00 – .02) | .46 (.39 – .54) | .13 (.10 – .16) | .33 (.28 – .39) | .01 (.00 – .03) | .52 (.47 – .58) |
| 2016 | Parity ≥ 3 | .15 (.09 – .20) | .35 (.28 – .42) | .01 (.00 – .02) | .49 (.41 – .58) | .12 (.08 – .15) | .36 (.30 – .41) | .01 (.00 – .02) | .52 (.46 – .58) |
| 2018 | Parity ≥ 3 | .17 (.13 – .22) | .39 (.32 – .45) | .01 (.00 – .03) | .42 (.34 – .51) | .11 (.08 – .14) | .35 (.29 – .40) | .01 (.00 – .02) | .54 (.49 – .58) |
| Δ (2018–2012) | Parity ≥ 3 | 0.01 | 0.02 | -0.01 | -0.02 | -0.01 | 0.05* | 0.00 | -0.04* |
| 2012 | Age 15–24 | .89 (.87 – .91) | .08 (.06 – .10) | .00 (.00 – .01) | .03 (.01 – .04) | .85 (.82 – .87) | .12 (.10 – .13) | .02 (.01 – .03) | .02 (.01 – .03) |
| 2014 | Age 15–24 | .85 (.83 – .88) | .10 (.08 – .12) | .00 (.00 – .01) | .04 (.02 – .05) | .84 (.82 – .86) | .13 (.12 – .15) | .01 (.00 – .01) | .02 (.01 – .03) |
| 2016 | Age 15–24 | .86 (.83 – .90) | .10 (.07 – .13) | .00 (.00 – .01) | .03 (.02 – .05) | .82 (.78 – .85) | .15 (.12 – .17) | .01 (.00 – .03) | .02 (.01 – .03) |
| 2018 | Age 15–24 | .83 (.80 – .86) | .11 (.09 – .13) | .01 (.00 – .01) | .05 (.03 – .07) | .81 (.78 – .84) | .16 (.13 – .19) | .01 (.00 – .02) | .01 (.01 – .02) |
| Δ (2018–2012) | Age 15–24 | -0.05* | 0.03* | 0.00 | 0.02 | -0.04 | 0.05* | -0.01 | -0.01* |
| 2012 | Age 25–34 | .65 (.62 – .69) | .22 (.18 – .26) | .01 (.00 – .01) | .12 (.09 – .14) | .52 (.49 – .55) | .28 (.25 – .31) | .01 (.01 – .02) | .19 (.15 – .22) |
| 2014 | Age 25–34 | .64 (.60 – .68) | .22 (.20 – .25) | .02 (.01 – .03) | .12 (.09 – .15) | .51 (.48 – .55) | .30 (.27 – .32) | .03 (.01 – .04) | .16 (.14 – .19) |
| 2016 | Age 25–34 | .65 (.61 – .69) | .22 (.19 – .25) | .01 (.00 – .02) | .12 (.09 – .15) | .55 (.51 – .60) | .27 (.24 – .31) | .02 (.01 – .02) | .16 (.12 – .19) |
| 2018 | Age 25–34 | .68 (.64 – .71) | .21 (.18 – .24) | .01 (.00 – .01) | .11 (.08 – .13) | .52 (.48 – .56) | .32 (.29 – .35) | .02 (.01 – .03) | .13 (.11 – .16) |
| Δ (2018–2012) | Age 25–34 | 0.03 | -0.01 | 0.00 | -0.01 | 0.00 | 0.04* | 0.01 | -0.05* |
| 2012 | Age 35–44 | .22 (.18 – .25) | .40 (.36 – .44) | .01 (.00 – .02) | .37 (.33 – .42) | .11 (.09 – .13) | .39 (.36 – .43) | .01 (.00 – .01) | .49 (.45 – .53) |
| 2014 | Age 35–44 | .22 (.19 – .25) | .38 (.33 – .42) | .02 (.01 – .03) | .39 (.34 – .43) | .14 (.12 – .17) | .43 (.40 – .47) | .02 (.01 – .03) | .41 (.37 – .44) |
| 2016 | Age 35–44 | .27 (.23 – .31) | .37 (.32 – .41) | .02 (.01 – .03) | .35 (.30 – .39) | .13 (.11 – .16) | .42 (.38 – .47) | .01 (.01 – .02) | .43 (.39 – .47) |
| 2018 | Age 35–44 | .26 (.22 – .29) | .40 (.36 – .43) | .02 (.00 – .03) | .33 (.29 – .37) | .13 (.10 – .15) | .39 (.36 – .42) | .01 (.01 – .02) | .47 (.43 – .50) |
| Δ (2018–2012) | Age 35–44 | 0.04* | 0.00 | 0.01 | -0.04* | 0.02 | 0.00 | 0.01 | -0.02* |

Note. NSFG data from 2011 to 2019; men and women between age 15 and 44. N = 39,487.

* p < .05 Test of equal proportions in 2012 and 2018.
